# Identification of the novel potential pathogen *Trueperella pecoris* with interspecies significance by LAMP diagnostics

**DOI:** 10.1038/s41598-023-40787-1

**Published:** 2023-08-27

**Authors:** Antonia Kreitlow, Siti Gusti Ningrum, Christoph Lämmler, Marcel Erhard, Christiane Hoffmann, Madeleine Plötz, Amir Abdulmawjood

**Affiliations:** 1 Institute of Food Quality and Food Safety, University of Veterinary Medicine Hannover, Bischofsholer Damm 15, 30173 Hannover, Germany; 2 Faculty of Veterinary Medicine, Universitas Wijaya Kusuma Surabaya, Jl. Dukuh Kupang XXV No.54, Dukuh Kupang, Surabaya, 60225 Indonesia; 3 Institute of Hygiene and Infectious Diseases of Animals, Justus Liebig University Giessen, Frankfurter Str. 87-89, 35392 Giessen, Germany; 4 RIPAC-LABOR GmbH, Am Mühlenberg 11, 14476 Potsdam, Germany

**Keywords:** Infectious-disease diagnostics, Applied microbiology, PCR-based techniques

## Abstract

*Trueperella pecoris* was described as a new species of the genus *Trueperella* in 2021 and might be pathogenic to various animal species. However, the lack of a suitable diagnostic test system stands in the way of epidemiological surveys to clarify possible causalities. In this study, a Loop-mediated Isothermal Amplification (LAMP) assay was developed and validated that was highly specific for *T*. *pecoris*. The assay provided an analytical sensitivity of 0.5 pg/25 µL and showed 100% inclusivity and exclusivity for 11 target and 33 non-target strains, respectively. Three different DNA extraction methods were evaluated to select the most LAMP-compatible method for cell disruption in pure and complex samples. Using an on-site applicable single-buffer DNA extraction with additional heating, the cell-based detection limit was 2.3 CFU/reaction. Finally, the LAMP assay was validated by means of artificially contaminated porcine lung tissue samples in which minimal microbial loads between 6.54 and 8.37 × 10^3^ CFU per swab sample were detectable. The LAMP assay established in this study represents a suitable diagnostic procedure for identifying *T. pecoris* in clinical specimens and will help to collect epidemiological data on the pathogenicity of this species.

## Introduction

In 2021, *Trueperella* (*T.*) *pecoris* was introduced as a novel species of the genus *Trueperella* by Schönecker et al.^1^. Strains were isolated from specimens of dairy cattle suffering from mastitis or abortion and of a dead pig showing fibrinous pleurisy and suppurative bronchopneumonia during necropsy. In addition to this bacterium, other potential pathogens were also co-isolated, so that an etiological link could not be proven, but could not be excluded either. Based on the anamnestic surveys and results of cultural examinations, the authors suspected at least a probable involvement of *T. pecoris* in the pathogenesis of the mastitis cases described in that study^1^. However, other species of the genus *Trueperella*, such as *T. abortisuis* and *T. pyogenes* have also been associated with pathological manifestations in pigs, including abortion or multi-organ inflammation^2,3^. Recently, another *T. pecoris* strain was isolated from the necrotic vestibulitis of a camel^4^.

Apart from the previously described origins of bacterial isolates and their phenotypic and genotypic characteristics, no information is available on this novel *Trueperella* species. Its distribution and pathogenic significance remain unclear, particularly as practicable, specific and reliable detection methods are lacking. Cultural examination followed by biochemical differentiation and sequencing of the 16S rRNA gene as described by Schönecker et al.^1^ may already offer possibilities for identifying *T. pecoris* but are very labour-intensive and time-consuming. To efficiently study the epidemiology of this potential pathogen, rapid diagnostic methods suitable for screening large numbers of heterogeneous specimens are necessary.

As detailed sequence information on various *T. pecoris* strains is available^1^, the development of molecular detection methods, for example based on Loop-mediated Isothermal Amplification (LAMP) should be considered. This technique was introduced in 2000^5^ and since then has gained a lot of attention in the scientific community. Compared to alternative methods like PCR, LAMP enables rapid and continuous amplification at a constant temperature, eliminating the need for an expensive and non-portable thermocycling device^6^. In addition, the method was shown to be highly specific and sensitive in detecting genomic targets while being robust to inhibitory components in various samples^7, 8^. Reactions can be carried out at low cost, for example using a heating block^9^. For the subsequent detection of LAMP products, various methods are available, which are often based on a visible color change or a generated turbidity^10^. In addition to low-cost options for performing this method, which can be of great benefit in limited laboratory environments, there are also portable real-time fluorometers or turbidimeters available on the market^11,12^. These offer additional features like real-time monitoring of the LAMP reaction or melting curve generation for evaluating the specificity of a LAMP product.

The advantageous characteristics of the LAMP method have led to the development of several assays intended as point-of-care diagnostic tools for a wide range of pathogens^13^. For the detection of microorganisms, LAMP is almost universally applicable as long as a specific genomic target region is available. Therefore, the present study aimed to develop and validate an easy-to-perform LAMP assay for detecting *T. pecoris* in clinical specimens. Primarily, it should provide a diagnostic tool for further research into epidemiological aspects and pathogenic significance of this new species. Additionally, the assay should meet the requirements for possible later use in patient diagnostics.

## Results

### LAMP assay setup

**Figure 1:**
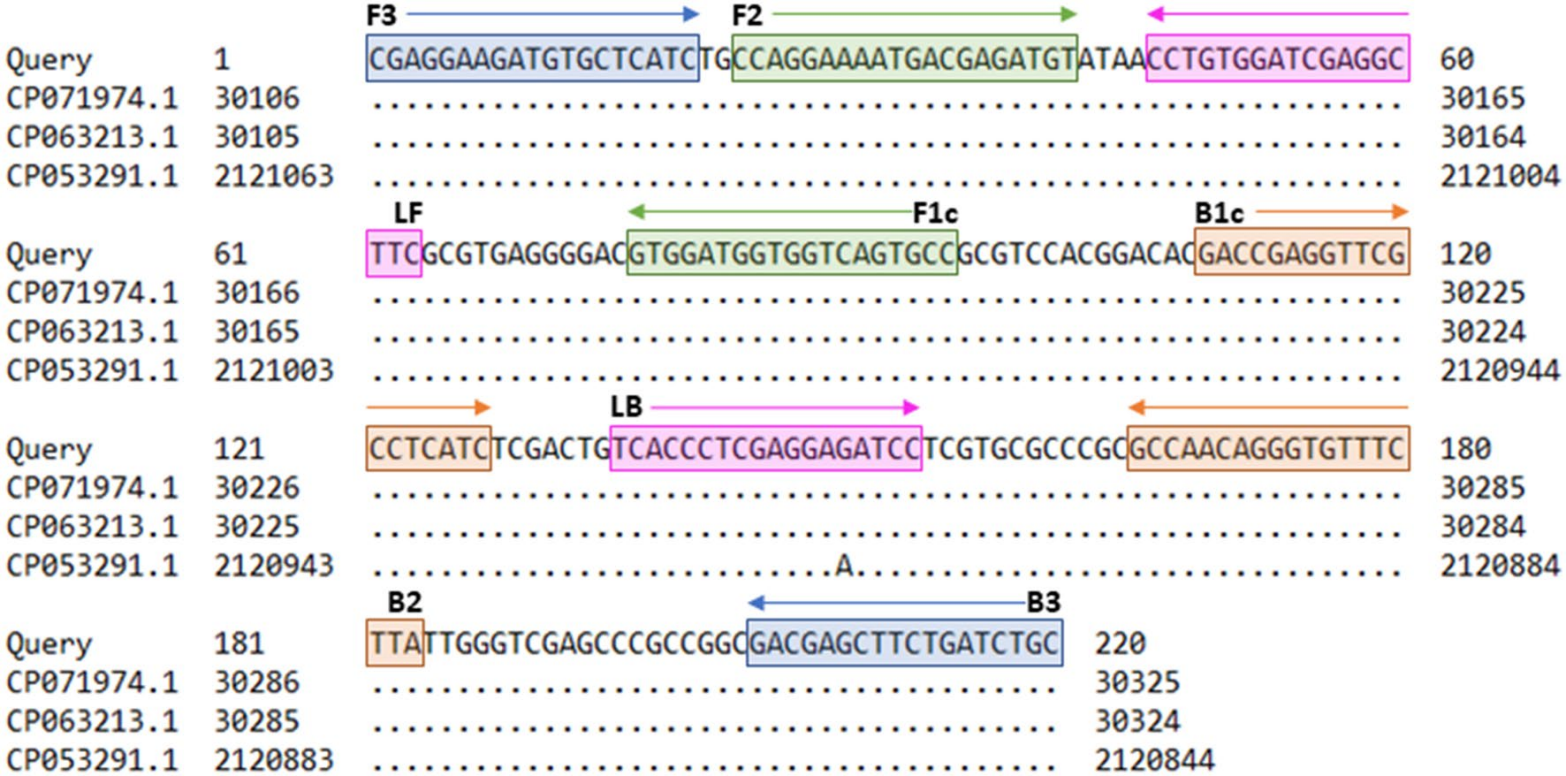
TP LAMP assay primers. LAMP primers were based on strain *T. pecoris* 19OD0592 (Acc. No. NZ_CP063212.1). Alignment with other *T. pecoris* genomes showed the high conservation of the target region. Query = *T. pecoris* strain 19OD0592, CP071974.1 = *T. pecoris* strain 15IMDO307, CP063213.1 = *T. pecoris* strain 15A0121, CP053291.1 = *T. pecoris* strain 19M2397.

**Figure 2:**
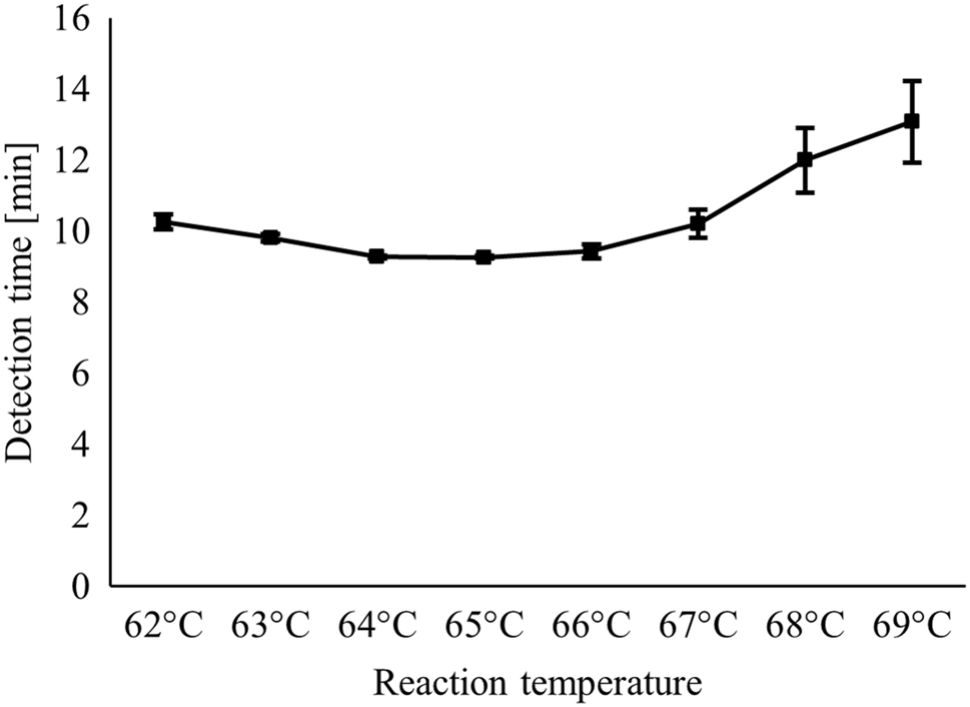
Temperature optimization of the TP LAMP assay. Detection times are shown for 0.5 pg positive control DNA/25 µL from strain *T. pecoris* DSM 111392^T^ as a function of the reaction temperature used. Marks and error indicator bars represent the mean and standard deviation, respectively, of three independent measurements.

A specific and conserved genomic target region within strain *T. pecoris* 19OD0592 (Acc. No. NZ_CP063212.1) was determined from 31,777 to 31,996 bp using the BLAST algorithm by the National Center for Biotechnology Information (NCBI) (https://blast.ncbi.nlm.nih.gov). Primer sequences and positions are shown in Fig. 1. For setting up the *Trueperella pecoris* (TP) LAMP assay, primer concentrations and reaction temperature were optimized. The concentrated TP primer set resulted in more effective amplification and gave an advantage of about 5 min in detection time compared to the standard TP primer set (data not shown). Using the concentrated primer set, the shortest detection times were measured at 65 °C (Fig. 2). Therefore, the concentrated primer set and the reaction temperature of 65 °C were selected as the final running parameters. The positive control had a mean detection time of 9.26 ± 0.03 min with a LAMP product melting temperature of 90.7 ± 0.06 °C. In the following experiments, LAMP product melting temperatures of 90.7 ± 1.0 °C were classified as specific for different isolates.

### Analytical specificity

The analytical specificity of the TP LAMP assay was evaluated by means of 44 bacterial strains including 11 T*. pecoris* isolates of different origins (Table 1) and 33 non-target species. Inclusivity and exclusivity of the assay were both 100% with respect to the isolates tested (Table 2).

**Table 1:** Origin of the *T. pecoris* strains used in this study.

Strain	Animal species	Sample matrix	Country	Verification of identity
*Trueperella pecoris* DSM 111392^T^	Heifer	Udder secretion	Switzerland	Schönecker et al.^1^
*T. pecoris* 203/7	Camel	Vestibulum	Germany	Ahmed et al.^4^
*T. pecoris* 3866/15	Cow	Uterus	Germany	16S rRNA gene sequencing
*T. pecoris* 6392/15	Cow	Swab inter udder eczema	Germany	16S rRNA gene sequencing
*T. pecoris* A110519	Cattle	Inner organ	Bulgaria	16S rRNA gene sequencing
*T. pecoris* A110567	Cattle	Inner organ	Netherlands	16S rRNA gene sequencing
*T. pecoris* A143782	Cattle	Inner organ	Netherlands	16S rRNA gene sequencing
*T. pecoris* A116983	Cattle	Inner organ	Netherlands	16S rRNA gene sequencing
*T. pecoris* A113576	Cattle	Inner organ	Netherlands	16S rRNA gene sequencing
*T. pecoris* A133311	Pig	Vaginal swab	Germany	16S rRNA gene sequencing
*T. pecoris* A120783	Cattle	Cervix	Germany	16S rRNA gene sequencing
*T. pecoris* A126428	Pig	Inner organ	Germany	16S rRNA gene sequencing

**Table 2:** Bacterial isolates used for testing inclusivity and exclusivity of the *TP LAMP assay*. ^*T*^ = Type strain; Origin of isolates: DSM = German Collection of Microorganisms and Cell Cultures GmbH, NCTC = National Collection of Type Cultures, *provided by RIPAC-LABOR GmbH—ENOVAT European strain database, not designated = inhouse culture collection.

Inclusivity testing	LAMP detection (min:s)	
*Trueperella pecoris* DSM 111392^T^	06:21	
*T. pecoris* 203/7	08:41	
*T. pecoris* 3866/15 (field strain)	06:26	
*T. pecoris* 6392/15 (field strain)	07:18	
*T. pecoris* A110519 (field strain)*	06:41	
*T. pecoris* A110567 (field strain)*	06:42	
*T. pecoris* A143782 (field strain)*	06:44	
*T. pecoris* A116983 (field strain)*	07:55	
*T. pecoris* A113576 (field strain)*	06:50	
*T. pecoris* A133311 (field strain)*	06:55	
*T. pecoris* A120783 (field strain)*	07:48	
*T. pecoris* A126428 (field strain)*	06:59	

Exclusivity testing	Number of isolates	
*Arcanobacterium hippocoleae* (incl. DSM 15539^T^ and field strains)	7	Negative
*A. phocae* (incl. DSM 10002^T^ and field strains)	5	Negative
*A. phocisimile* (DSM 26142^T^)	1	Negative
*A. pinnipediorum* (incl. DSM 28752^T^ and field strain)	2	Negative
*A. pluranimalium* (DSM 13483^T^)	1	Negative
*A. buesumense* (incl. DSM 112952^T^ and field strain)	2	Negative
*Escherichia coli* (incl. DSM 1103, DSM 22665, DSM 22316, DSM 22311)	4	Negative
*Enterococcus faecalis* (incl. DSM 13591, NCTC 8727)	2	Negative
*E. faecium* (incl. DSM 25389, DSM 25390)	2	Negative
*Trueperella abortisuis* (DSM 19519^T^)	1	Negative
*T. bernardiae* (DSM 9152^T^)	1	Negative
*T. bialowiezensis* (DSM 17162^T^)	1	Negative
*T. pyogenes* (incl. DSM 20630^T^ and field strains)	4	Negative

### Analytical sensitivity

**Figure 3:**
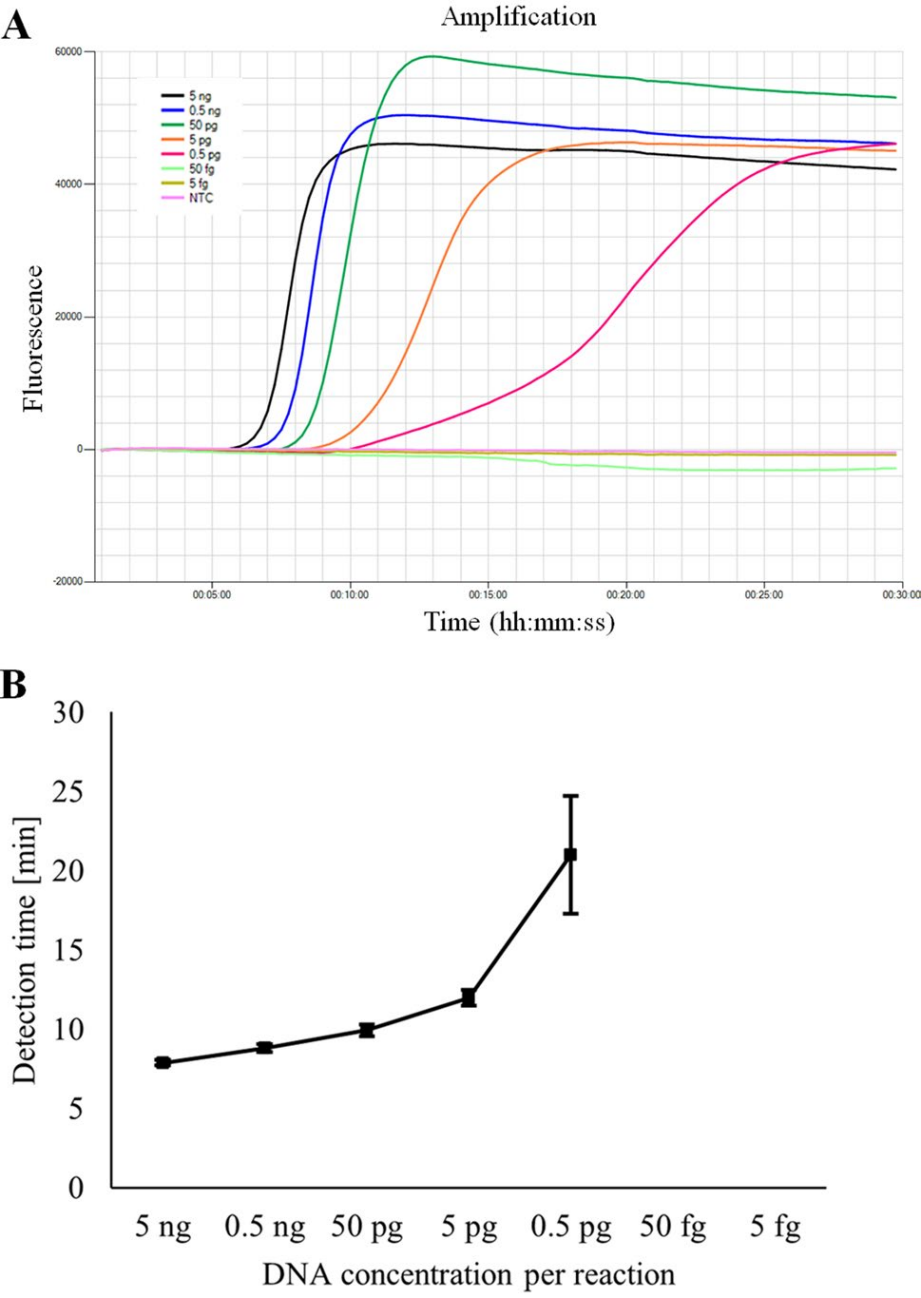
Analytical sensitivity of TP LAMP assay. (**A**) Amplification curves for ten-fold serially diluted DNA of *T. pecoris* DSM 111392^T^. Characteristic for LAMP, the amplification curves flattened with decreasing DNA concentration. NTC = non-template control. (**B**) Detection times for ten-fold serially diluted DNA of *T. pecoris* DSM 111392^T^ as a function of DNA concentration per reaction. Marks and error indicator bars represent the mean and standard deviation, respectively, of three independent measurements. With decreasing DNA concentration, detection times did not increase linearly. For this reason, LAMP was not suitable for quantifying the target species.

To determine the analytical sensitivity of the TP LAMP assay, ten-fold serially diluted DNA was tested. A minimum concentration of 0.5 pg DNA/25 µL could be detected in all three repetitions. Decreasing DNA content in the reaction mixture was associated with a flattening of the amplification curves and a non-linear increase in detection times (Fig. 3A + B). In contrast, a real-time PCR targeting the same amplicon as TP LAMP detected a minimum concentration of 50 fg DNA/25 µL in all three repetitions.

### Cell-based detection limits

Determining the cell-based detection limits of the TP LAMP assay was based on three DNA extraction methods. Using the DNeasy Blood & Tissue kit, up to 6.0 × 10^2^ CFU/25 µL were detectable by TP LAMP in all three repetitions. This was 600 × less sensitive compared to the genomic target-identical real-time PCR, which was able to detect up to 1 CFU/20 µL. The detection limit of the TP LAMP assay improved to 2.3 × 10^2^ CFU/25 µL using single-buffer extraction and even reached 2.3 CFU/25 µL after an additional heating step was applied.

### Detection limits in artificially contaminated porcine lung tissue samples

Artificially contaminated porcine lung tissue samples were tested to evaluate the application suitability of the TP LAMP assay under the influence of sample material and background flora of the specimen type. The lung tissue was collected during dissections performed as a part of routine diagnostic procedures at the Field Station for Epidemiology in Bakum (University of Veterinary Medicine Hannover). Detection limits for *T. pecoris* strains DSM 111392^T^ and 203/7 were determined and assessed using three swab sampling adapted DNA extraction methods. Corresponding results in terms of pathogen count per swab are shown in Table 3. Using kit-extracted DNA, TP LAMP was 100- and 300-fold less sensitive than real-time PCR for *T. pecoris* strains DSM 111392^T^ and 203/7, respectively. However, all results could be clearly interpreted and no false negatives occurred when samples were tested with TP LAMP. In contrast, real-time PCR frequently produced false positive signals under the influence of the sample matrix. Melting curves of the LAMP products were always uniform, whereas melting curves of the corresponding PCR products showed a wide variety of profiles compared to the positive control, with mostly diverging but also partly uniform melting peaks.

**Table 3:** Detection limits of the TP LAMP assay in artificially contaminated porcine lung tissue samples. Cell counts are indicated as bacterial load per swab.

	*T. pecoris* strain DSM 111392^T^	*T. pecoris* strain 203/7
Kit-extraction	1.87 × 10^4^ CFU	1.05 × 10^4^ CFU
LPTV buffer	6,57 × 10^4^ CFU	8,37 × 10^5^ CFU
LPTV buffer + heat	6,57 × 10^3^ CFU	8,37 × 10^3^ CFU

## Discussion

*Trueperella pecoris* is a novel bacterial species that appears to be related to several animal diseases, including mastitis in cattle, bronchopneumonia in pigs, and impairments of the reproductive tract in ruminants^1,4^. In addition, the presence of *T. pecoris* in the bovine mammary gland suggests that human infection through consumption of contaminated milk should also be considered, as the human pathogenic properties of *Trueperella* spp. are frequently described in the literature^14, 15^ However, the available data do not allow definitive conclusions to be drawn as to whether *T. pecoris* actually occurs as a disease-causing agent. In this study, a detection system was developed that enables specific diagnostics in clinical specimens and thus helps to generate epidemiological data and to clarify the pathogenic significance of the bacterium.

Primers of the TP LAMP assay were based on a gene region encoding an S9 family peptidase that has been shown to be unique in *T. pecoris* by BLAST analysis. The functions of these enzymes have so far been best studied in humans and are associated with complex immunological and hormonal metabolic processes^16^. It can be assumed that the targeted S9 family peptidase also plays a key role in the metabolism of *T. pecoris* and that the coding gene is constantly present and conserved in the bacterial genome. This was reflected in the high level of inclusivity of the TP LAMP assay for target species and exclusivity for non-target species.

Performances of TP LAMP and PCR targeting the same amplicon were compared in terms of analytical sensitivity. In the literature, there is true competition between the two amplification techniques, often emphasizing that LAMP is superior to PCR^10^. However, this must be viewed in a differentiated manner. For example, LAMP usually achieves a higher analytical sensitivity only when compared to conventional PCR^17, 18^. Considering results of real-time PCR, similar or even worse detection limits are frequently obtained^19,20^. Therefore, it was not unusual that the TP LAMP assay presented in this study showed an analytical sensitivity 10 times lower than that of real-time PCR. The TP LAMP assay was able to compensate for this difference with other advantageous properties and was superior to real-time PCR, particularly in the testing of complex sample matrices.

When examining the cell-based detection limit, interestingly, there was a significant loss in sensitivity when the kit-extracted DNA was tested. In contrast, the performance of the TP LAMP assay could be improved by LPTV buffer extraction with additional heating, which was most likely due to a comparatively higher DNA yield. In addition, this DNA extraction method is characterized by its rapidity and its suitability for on-site use. However, no washing steps are included, meaning that the inhibitory components are not removed. This regularly poses a problem for PCR diagnostics, as insufficiently processed DNA templates of low quality can be associated with suppression of amplification and obtaining false negative results^21^. In contrast, LAMP is known for its ability to deal with potential inhibitors^22^, making the method compatible with non-sophisticated, fast, and low-cost DNA preparation techniques.

The TP LAMP assay also demonstrated the previously described properties when tested on porcine lung tissue samples. This matrix was selected as it may represent a potential target organ for infection with *T. pecoris*^1^*.* Both tissue particles and blood adhered to the swab samples taken, thus containing several inhibitory components like hemoglobin, lactoferrin, or immunoglobulin G^23,24^. Similar to the previous findings of this study, TP LAMP showed lower sensitivity for kit-extracted DNA samples compared to real-time PCR, but compensated for this limitation when templates from LPTV buffer extraction with subsequent heating were used. However, differences of TP LAMP and real-time PCR regarding sensitivity in complex samples can only be evaluated to a limited extent, as false positive reactions frequently occurred in real-time PCR examination. In contrast, all TP LAMP reactions could be clearly interpreted and showed no false positive results, underpinning the robustness of this assay.

Currently, no cultural or biomolecular reference method is available for the diagnosis of *T. pecoris*, so that the identification of the bacterium is so far only possible via the non-directed collection of suspicious isolates followed by laborious and time-consuming sequence analyses. Consequently, securing suitable sample material from infected animals during appropriate studies and subsequent testing by TP LAMP would therefore be required to finally complete the validation of this assay. Since only the 11 *T. pecoris* strains listed in Table 1 have been isolated to date, this process is expected to take a longer period of time and could not be addressed in this study. No knowledge is yet available regarding the zoonotic potential of this species, but if transmission to humans occurs through food-producing animals, validation of the TP LAMP assay should also be extended to meat and milk samples.

In conclusion, the TP LAMP assay described in this study represents the first available method for diagnosing *T. pecoris* infections in complex sample materials. The fact that the assay detects the bacterium with high specificity and sensitivity in non-purified templates reduces the costs, labor, and time for sample analysis, while at the same time ensuring high reliability of the results. These are suitable conditions for conducting large-scale prevalence studies, since the TP LAMP assay can be used under both laboratory and field conditions. The data that can now be collected using TP LAMP diagnostics can be processed to draw conclusions about the pathogenicity of *T. pecoris* in humans and animals, which should be the subject of future studies.

## Materials and methods

### LAMP primer

The sequence of *T. pecoris* strain 19OD0592 served as the basis for designing six specific LAMP primers using LAMP designer software version 1.15 (PREMIER Biosoft, Palo Alto, CA, USA). Sequence data was obtained from the GenBank database (acc. No. NZ_CP063212.1) provided by NCBI (Bethesda, USA). For determining a species-specific sequence region, sections of the *T. pecoris* genome were aligned successively with other sequence data available at GenBank using the megablast algorithm of the integrated Basic Local Alignment Search Tool (BLAST) (NCBI). After importing the selected data into the LAMP designer software, various LAMP primers were generated using default parameters and subjected to the integrated primer BLAST^25^. Subsequently, an appropriate primer set, consisting of four basic and two loop primers, was selected based on the ranking by the software and according to optimal specificity properties (Fig. 1). Production of the LAMP primers was contracted to Eurofins Genomics GmbH (Ebersberg, Germany).

### LAMP assay optimization and setup

All LAMP reactions were carried out using the real-time fluorometer Genie II^®^ by OptiGene Ltd. (Horsham, UK). The device is equipped with a rechargeable battery and can be operated on site via touchscreen, which also allows direct result visualization. To determine the optimal composition of the individual TP LAMP primers in a reaction mixture, two primer sets, consisting of a standard and a concentrated version, were prepared in accordance with the recommendations of OptiGene Ltd. Using the standard TP primer set, concentrations of primers F3 and B3, primers FIP and BIP, and primers LF and LB in a reaction mixture were 0.2 µM, 0.8 µM, and 0.4 µM, respectively. In contrast, concentrations of primers F3 and B3, primers FIP and BIP, and primers LF and LB in a reaction mixture were 0.2 µM, 2.0 µM, and 1.0 µM, respectively, when the concentrated TP primer set version was used. Each reaction mixture with a total volume of 25 µL contained 15 µL GspSSD Isothermal Mastermix (ISO-001) (OptiGene Ltd.), 2.5 µL primer mix, 2.5 µL nuclease-free water (NFW), and 5 µL DNA template. The optimal primer set was selected by detection times achieved for standardized DNA (0.1 ng/µL) from *T. pecoris* reference strain DSM 111392^T^ and *T. pecoris* field strain 203/7. Subsequently, the reaction temperature of the TP LAMP assay was optimized for the selected primer set by evaluating standardized DNA (0.1 ng/µL) from *T. pecoris* reference strain DSM 111392^T^ using a temperature gradient between 62 and 69 °C on the Genie II^®^ instrument. The temperature at which the lowest detection times were measured was applied for subsequent LAMP tests. Optimization experiments were performed in triplicate and evaluated with the Genie Explorer version 2.0.7.11 application software (OptiGene Ltd.) using default parameters. Each run included a positive and negative control reaction using *T. pecoris* DSM 111392^T^ DNA template (0.1 ng/µL) and NFW, respectively.

### Real-time PCR assay

A real-time PCR assay targeting the same amplicon as the TP LAMP assay was established to compare the performance of both amplification techniques. For this purpose, LAMP primers TP-F3 and TP-B3 were used as forward and reverse primer in the real-time PCR assay, respectively. Each reaction had a total volume of 20 µL and contained 10 µL FastStart Essential DNA Green Master (Roche), 1 µL of both TP-F3 and TP-B3 primer (10 µM), 3 µL H_2_O, and 5 µL DNA template. Runs started with initial denaturation at 95 °C for 10 min, followed by 45 cycles of three-step amplification, and melting. Three-step amplification included denaturation at 95 °C for 10 s, annealing at 60 °C for 10 s and elongation at 72 °C for 10 s with ramp rates of 4.4, 2.2 and 4.4 °C. respectively. Fluorescence signals were measured after each elongation step. Melting curves were generated using a temperature–time profile of 95 °C for 10 s, 65 °C for 60 s and 97 °C for 1 s. As with the TP LAMP assay, *T. pecoris* DSM 111392^T^ DNA template (0.1 ng/µL) and NFW were used as positive and negative control, respectively, in each run.

### DNA extraction from bacterial isolates

For DNA extraction, all bacteria were grown on Columbia sheep blood agar (Oxoid Deutschland GmbH, Wesel, Germany) in accordance with species-specific requirements. *Escherichia coli*, *Enterococcus faecalis* and *Enterococcus faecium* isolates were incubated for 24 h at 37 °C under aerobic conditions. In contrast, a candle jar atmosphere was used to cultivate *Trueperella* spp. and *Arcanobacterium* spp. for 24–48 h at 37 °C. For isolation of DNA, the DNeasy Blood & Tissue Kit (Qiagen GmbH, Hilden, Germany) was used with slight modification. Instead of harvesting bacterial cells from liquid cultures by centrifuging, 5–10 colonies of Gram-positive or Gram-negative bacteria were picked from agar plates and directly resuspended in enzymatic lysis buffer (20 mM Tris–HCl, pH 8.0; 2 mM sodium EDTA; 1.2% Triton^®^ X-100; lysozyme, 20 mg/mL) or ATL buffer, respectively. The following steps for DNA extraction were performed in accordance with the manufacturer's instructions.

### Analytical specificity

A total of 44 bacterial isolates were used to test the analytical specificity of the TP LAMP assay (Table 2). Strains were derived from the inhouse culture collection of the Institute of Food Quality and Food Safety, University of Veterinary Medicine Hannover, Foundation (Hannover, Germany), the German Collection of Microorganisms and Cell Cultures GmbH (Braunschweig, Germany), the National Collection of Type Cultures (Salisbury, UK), and from the RIPAC-LABOR GmbH (Potsdam, Germany, ENOVAT European strain database). For inclusivity testing, previously described *T. pecoris* strains originating from cattle and camel^1,4^ as well as further *T. pecoris* isolates were considered (Table 1). Positive results were defined by the appearance of a fluorescence signal associated with a specific melting temperature. Strains used for exclusivity testing were selected due to their close genetic relationship with *T. pecoris* and their coexistence in the same environment.

### Analytical sensitivity

The analytical sensitivity of the TP LAMP assay was determined by means of ten-fold serially diluted DNA from strain *T. pecoris* DSM 111392^T^. AE buffer from the DNeasy Blood & Tissue Kit (Qiagen) was used as dilution medium. DNA concentrations were adjusted to 1 ng/µL to 1 fg/µL and each template was tested in triplicate using TP LAMP. For comparison, each template was also examined three times by real-time PCR. The analytical sensitivity was defined as the minimum DNA amount per reaction at which results were positive in all three replicates.

### DNA extraction from cells and porcine lung tissue

Three different DNA extraction methods, including a kit-based method for obtaining highly purified DNA as well as a single-buffer extraction and a single-buffer extraction with heating, both adapted for on-site application, were used to isolate DNA from *T. pecoris* cell suspensions and porcine lung tissue swab samples. Kit-based extraction was performed using the DNeasy Blood & Tissue kit (Qiagen) in accordance with the manufacturer’s recommendations. The required incubation steps at 37 °C and 56 °C lasted 1 h each. To compare the sensitivity of LAMP and PCR, all kit-based isolates were tested with both methods. Applying the single buffer extraction method, pellets were suspended in 500 µL LPTV buffer (AmplexDiagnostics GmbH, Gars am Inn, Germany), incubated for 5 min at room temperature and directly tested exclusively using TP LAMP. Subsequently, the same LPTV cell suspensions were subjected to heating at 100 °C for 10 min and tested again.

### Cell-based detection limit

The cell-based detection limit of the TP LAMP assay was determined by ten-fold serially diluted cell-suspensions. For this purpose, strain *T. pecoris* DSM 111392^T^ was grown for 48 h at 37 °C in a candle jar. Colony material was suspended in 5 mL NaCl solution (0.9%) until a turbidity of 2.0 McFarland units (MFU) was achieved. Subsequently, the suspension was ten-fold serially diluted from 10^–1^ to 10^–8^ using 9-mL Maximum Recovery Diluent tubes (Oxoid Deutschland GmbH). DNA was extracted as previously described from cell pellets obtained from 1 mL of each dilution level after centrifuging the 1-mL aliquots for 10 min at 7500 rpm. Supernatants were carefully discarded. Each isolation procedure was repeated three times. The detection limit was defined as the minimum *T. pecoris* cell count at which results were positive in all three replicates.

### Artificial contamination of porcine lung tissue samples

For evaluating the suitability of the TP LAMP method under the influence of sample matrix and its background flora, swab samples were taken from porcine lung tissue and artificially contaminated with *T. pecoris*. Strain *T. pecoris* DSM 111392^T^ and the camel-associated *T. pecori*s strain 203/7 were used for artificial contamination. For this purpose, strains were grown for 48 h at 37 °C in a candle jar, and decimal dilution series were prepared as previously described. The surface of the lung of a clinically healthy pig as well as deeper tissue layers opened by a sterile incision into the lung tissue were dabbed with a sterile cotton swab. Subsequently, the swab was transferred into a 1.5-mL reaction tube filled with 900 µL MRD, repeatedly revolved, and finally squeezed out on the wall. For each strain, 10 tubes were prepared. From each dilution level of the two strain cell suspensions, 100 µL were taken and added to the swab material containing MRD medium. One tube per strain remained non-inoculated and served as a negative extraction control. After centrifuging the tubes at 7500 rpm for 10 min, supernatants were discarded carefully. Pellets were used for DNA extraction as previously described. The experiment was repeated three times on three consecutive days. The detection limit in porcine lung tissue samples was defined as the average *T. pecoris* cell count per swab at which results were positive in all three replicates.

### Data processing and statistical analyses

Data generated during the LAMP and PCR runs were analyzed using the Genie Explorer version 2.0.7.11 and Light Cycler^®^ 96 software version 1.1.0.1320 application software provided by the respective instrument manufacturers. Raw data processing and statistical analyses were performed with Microsoft Excel version 2303.

## Data Availability

The datasets used and analysed during the current study are available from the corresponding author on reasonable request.
